# The Impact of the COVID‐19 Pandemic on Mental Health and Cognitive Function in Patients With Cancer: A Systematic Literature Review

**DOI:** 10.1002/cnr2.70008

**Published:** 2024-10-23

**Authors:** Sílvia Almeida, Diana Frasquilho, M. Teresa Cordeiro, Teresa Neto, Berta Sousa, Fátima Cardoso, Albino J. Oliveira‐Maia

**Affiliations:** ^1^ Champalimaud Research and Clinical Centre Champalimaud Foundation Lisbon Portugal; ^2^ Graduate Programme in Clinical and Health Psychology Faculdade de Psicologia da Universidade de Lisboa Lisbon Portugal; ^3^ Breast Unit, Champalimaud Clinical Centre Champalimaud Foundation Lisbon Portugal; ^4^ NOVA Medical School, Faculdade de Ciências Médicas NMS, FCM, Universidade NOVA de Lisboa Lisbon Portugal

**Keywords:** anxiety, cancer, COVID‐19 pandemic, depression, mental health, systematic review

## Abstract

**Background:**

The COVID‐19 pandeminc has had widespread impacts, but its specific effects on mental health and cognitive function in patients with cancer remain under‐explored.

**Recent Findings:**

Data from the general population has suggested that mental health problems were frequent during the pandemic, namely during the initial stage of the outbreak. For patients with cancer, a systematic review and meta‐analysis of data published until January 2021 also showed elevated prevalence of depression and anxiety, and suggested that anxiety was more frequent than in health workers and healthy controls.

**Objective:**

This systematic review aimed to synthesize existing evidence on the impact of the COVID‐19 pandemic on mental health and cognitive function in patients with cancer.

**Methods:**

Studies were identified through systematic search of three electronic bibliographic databases (PubMed, Web of Science, and EBSCOHOST) with adapted search strings. We included only peer‐reviewed, nonqualitative, original research papers, published between 2019 and 2022, and reporting on mental health and/or cognition outcomes during the COVID‐19 pandemic in adult patients with cancer.

**Results:**

Of 3260 papers identified, 121 full text articles were retrieved and 71 met inclusion criteria. We found that patients with cancer reported high levels of psychological distress, anxiety and depression, as well as cognitive complaints during the pandemic. However, studies were not consistent in identifying these symptoms as effects of the pandemic specific for this population. In fact, longitudinal studies did not find consistent differences between pre‐ and post‐pandemic periods and, globally, patients with cancer did not report increased severity of these mental health symptoms in relation to the general population.

**Conclusion:**

Overall, while the COVID‐19 pandemic may have raised mental health challenges for patients with cancer, the diagnosis of cancer and associated treatments seemed to remain the main source of concern for these patients.

## 1 Background

There are strong reasons to believe that the COVID‐19 pandemic caused significant harm for the mental wellbeing of some populations [1], with particular concern for those where mental disorders were already highly prevalent. Patients with cancer are particularly vulnerable, given the high prevalence of mental health issues in this population [2]. Empirical knowledge about the links between the COVID‐19 pandemic and mental health is nevertheless still limited. For that reason, this systematic review aims to synthesize the literature on the impact of the COVID‐19 pandemic on mental health and cognitive function in patients with cancer.

A systematic review conducted early in the pandemic [3] emphasized that the general population revealed lower psychological well‐being, with higher scores of anxiety and depression when compared with before the COVID‐19 pandemic, and with specific concerns raised namely for patients with COVID‐19 and preexisting psychiatric disorders, as well as health workers. Moreover, several factors were associated with higher risk of psychiatric symptoms and/or low psychological well‐being, including being a woman, having poor self‐rated health and having relatives with COVID‐19. Indeed, a more recent meta‐review of prevalence meta‐analyses suggested that the prevalence of mental health problems in the general population during the pandemic was high, ranging from 20% to 36% [4]. Other studies have highlighted that while the general population experienced considerable psychological distress during the initial stage of the COVID‐19 outbreak, namely regarding symptoms of anxiety, depression, and posttraumatic symptoms, severe symptoms were reported by a minority [5].

Nonetheless, the increase in mental health problems during the pandemic is not universally agreed upon. A recent systematic review and meta‐analysis of longitudinal studies found that the rates for mental health problems peaked in April and May 2020, with anxiety and depression symptoms decreasing thereafter [6]. A more recent systematic review and meta‐analysis of 134 cohorts of participants from the general population showed that general mental health and anxiety symptoms did not change significantly from the prepandemic to the pandemic period, while depression symptoms increased minimally, particularly in women [7].

Nevertheless, concern remains regarding vulnerable groups, include those with preexisting mental or physical health issues, and those at greater risk of becoming mentally unwell in response to anxiety and loneliness surrounding the pandemic [1, 3, 5, 8]. Patients with cancer not only have physical health issues that make them especially vulnerable to becoming seriously ill or even dying of COVID‐19 [9, 10], they also have significantly higher rates of depression and anxiety relative to the general population [2, 9, 10, 11]. In fact, a systematic review and meta‐analysis of past‐year prevalence of depression and anxiety suggested that more than 20% and 10% of patients with cancer are affected, respectively, compared with 5% and 7% of the general population [2].

Additionally, cognitive dysfunction is also a common complaint among patients with cancer, with memory, processing speed, attention, and executive functions as the cognitive domains thought to be most impaired after chemotherapy [12]. Evidence was obtained primarily from studies on breast cancer patients due to the extensive body of research available for this population, providing a robust foundation for a better understanding of cancer‐related cognitive impairment, which can then be compared and contrasted with findings in other cancer types [12]. While the mechanisms involved in cancer‐related cognitive impairment remain unclear, cognitive dysfunction may be associated with adverse mental health outcomes [13], which may be of particular relevance in the pandemic context. In the general population, the lockdown restrictions in Italy have shown a significant effect on subjective cognitive functioning, particularly in everyday tasks involving attention, temporal orientation, and executive functions [14].

Despite the critical need to understand the impact of the COVID‐19 pandemic on the mental health of patients with cancer, comprehensive publications systematically synthesizing and critically appraising these questions over the entire timeframe of the pandemic are still lacking. A systematic review and meta‐analysis conducted by Ayubi, Bashirian, and Khazaei, which included articles published until January 2021, showed an overall prevalence of depression and anxiety of 37% and 38%, respectively [15]. Additionally, based on a meta‐analysis of only four studies, the authors suggested that patients with cancer had higher anxiety levels compared with controls (nurses and healthy controls).

Here we aimed to provide a more updated and comprehensive summary of the literature on the impact of the COVID‐19 pandemic on mental health and cognitive function, and their association, in patients with cancer. Specifically, we planned to compare pre‐ and during‐pandemic data regarding psychological distress, anxiety, depression and cognition of patients with cancer. Using the principles of the Population, Exposure, Comparator and Outcome (PECO) framework [16], this systematic review thus proposed to answer the following question: What are the expected effects of the COVID‐19 pandemic (E) on mental health and cognitive function outcomes (O) in patients with cancer (P), when compared with a previous state of nonexistence of the pandemic (C)? Understanding the impact of the COVID‐19 pandemic on the mental health of patients with cancer is essential not only to guide patient care but also for development of strategy for delivery of healthcare during future global crises. This study thus intends to offer comprehensive insights into the mental and cognitive health of patients with cancer during the COVID‐19 pandemic, to highlight risk factors associated with any deleterious effects, and to guide the development of targeted interventions and support systems to reduce the psychological burden on this patient population during public health emergencies.

## 2 Methods

This review followed the Preferred Reporting Items for Systematic review and Meta‐Analyses (PRISMA) Guidelines and PRISMA‐P checklist [17, 18]. The protocol for the review was preregistered with PROSPERO (ID CRD42020225313). A standardized electronic database search was performed using syntax based on Medical Subject Headings (MeSH), whenever possible. To improve sensitivity, the search was performed in three electronic bibliographic databases (PubMed, Web of Science, and EBSCOHOST) with the following search terms: (“mental” OR “psychology” OR “psychological” OR “psychiatry” OR “psychiatric” OR “anxiety” OR “anxious” OR “GAD” OR “panic” OR “depression” OR “depressive” OR “mood” OR “dysthymia” OR “MDD” OR “PTSD” OR “post‐traumatic stress disorder” OR “trauma” OR “stress” OR “adjustment” OR “distress” OR “suicide” OR “self‐harm” OR “parasuicide” OR “cognition” OR “cognitive” OR “cognitive disorders” OR “cognitive impairment” OR “cognitive dysfunction” OR “cognitive function” OR “cognitive domains” OR “neurocognitive disorders” OR “neurological” OR “neurology” OR “neuropsychology” OR “neurologic oncology” OR “neuro‐oncology” OR “neuropsychological” OR “memory” OR “attention” OR “concentration” OR “processing speed” OR “executive functions”) AND (“cancer” OR “tumour” OR “malignant” OR “neoplasia” OR “oncology” OR “oncological”) AND (“COVID” OR “COVID‐19” OR “coronavirus” OR “corona” OR “SARS‐Cov2” OR “pandemic”). The search was limited by language (English, Portuguese, Spanish, or French), and to research published between 2019 and 2022. Comprehensive searches were conducted until February 2022. All records were deduplicated using a citation tool (Zotero). Additionally, we manually screened the reference list of eligible articles to identify additional papers missed by the previous steps.

The inclusion criteria for this systematic review were as follows: adults (≥18 years old) with a current or past diagnosis of cancer; conducted during the COVID‐19 pandemic; having an assessment of anxiety, depression, posttraumatic symptoms, and cognition according to our outcomes of interest; and using valid questionnaires to assess these outcomes. All peer‐reviewed, nonqualitative observational study designs (i.e., cross‐sectional, cohort, case–control studies) and ecological studies reporting on associations between mental health or cognition outcomes and the COVID‐19 pandemic in adult patients with cancer were considered eligible. The exclusion criteria were experimental studies (i.e., intervention studies), published conference proceedings, conference abstracts, theses, literature reviews, and meta‐analyses. Studies focusing only on the general population, children, adolescents, or including animals were also excluded. Articles were excluded if they did not meet the inclusion criteria or did not report relevant findings. Results from the searches performed in PubMed, Web of Science, and EBSCO Host were deduplicated using a citation tool (Zotero). The resulting citations were reviewed separately by two graduate‐level researchers with expertise in mental health and oncology, and with knowledge in systematic literature review processes, for the selection of eligible articles based on titles. During the title screening phase, we identified very few longitudinal studies. Consequently, while the main objective of this study was to compare mental health outcomes of patients with cancer before and during the COVID‐19 outbreak, we decided to also include cross‐sectional and case–control studies to ensure a comprehensive analysis of the impact of the pandemic on mental health and cognitive outcomes in patients with cancer. At the following steps, the two researchers reviewed abstracts and full texts independently according to inclusion and exclusion criteria. At any step, when there was disagreement among the reviewers, it was discussed and resolved by consensus, with arbitration by a third reviewer when consensus was not possible. For reports in the final full paper consensus, we reviewed their respective reference lists for any additional relevant papers and for these we repeated the title, abstract, and full text review process as described above.

A data extraction form was designed a priori, pilot‐tested by the researchers on a small subset of studies, and adapted as needed to improve usability. Data extraction was completed independently by the two researchers. When consensus was not reached at comparison, disagreements were resolved by consensus and third‐person arbitration, as described above for study selection. The extraction form provided an overview of the key elements (sample, methodologies, results, etc.) from the eligible studies and supported comparison between studies to summarize data for the discussion. The variables extracted were organized in several themes, as described here. *Study identifiers* included author's names; study title; publication type; publication date; journal, volume, issue, and page numbers of publication; place of publication (i.e., first author's institutional address); and digital object identifier. *Study design* included design; time frame of study; country of study; and region of study (when reported). *Participants* included definitions of the target and source populations; size of population; and size of the source population. Relevant demographic information (e.g., age, sex) were extracted when reported. *Exposure information* included the name, date, and measurement method of parameters related to the COVID‐19 pandemic. *Outcome information* included information on the type of outcome (common mental health disorders, general mental health status, psychological distress, suicide, trauma, or cognitive functioning), the respective measurement method and, when applicable, the cut‐off score used for interpretation (self‐reported questionnaire, diagnostic interview etc.). *Analytical methods*, reflected the measure(s) of precision for the associations (e.g., standard error of the mean, standard deviation, and/or confidence intervals), extracted when provided. *Confounder variables*, that is, variables potentially affecting the association between the parameters related to the COVID‐19 pandemic and the outcomes of interest in patients with cancer, were extracted, as well as main strengths, limitations, and missing information. Additionally, we documented the recruitment period of each study. The studies were then classified according to the phases of COVID‐19 during which they took place, namely:

Early Phase (E): January 2020–June 2020. This phase includes the initial outbreak and global spread of COVID‐19, marked by the first wave of infections, widespread lockdowns, and the initial implementation of public health measures.

Middle Phase (M): July 2020–December 2020. This phase captures the period when initial restrictions were eased in many regions, followed by the emergence of subsequent waves of infection. It also reflects the period when people started adapting to the new lifestyles, and the impact of prolonged public health measures became evident.

Late Phase (L): January 2021–June 2022. This phase includes the rollout of vaccination programs, the emergence of new variants, and varying levels of restrictions and public health measures across different regions.

The methodological quality of the studies was assessed using the Quality Assessment Tool for Quantitative Studies (National Collaborating Centre for Methods and Tools, 2008) [19]. This is a 19‐items checklist, assessing 8 methodological domains: selection bias, study design, confounders, blinding, data collection methods, withdrawals and dropouts, intervention integrity, and analyses. A global rating was determined based on the scores of each component. Two researchers rated the studies in each domain and for the overall quality. Discrepancies were solved by consensus with third‐person arbitration if necessary.

## 3 Results

### 3.1 Characteristics of the Included Studies

**FIGURE 1:**
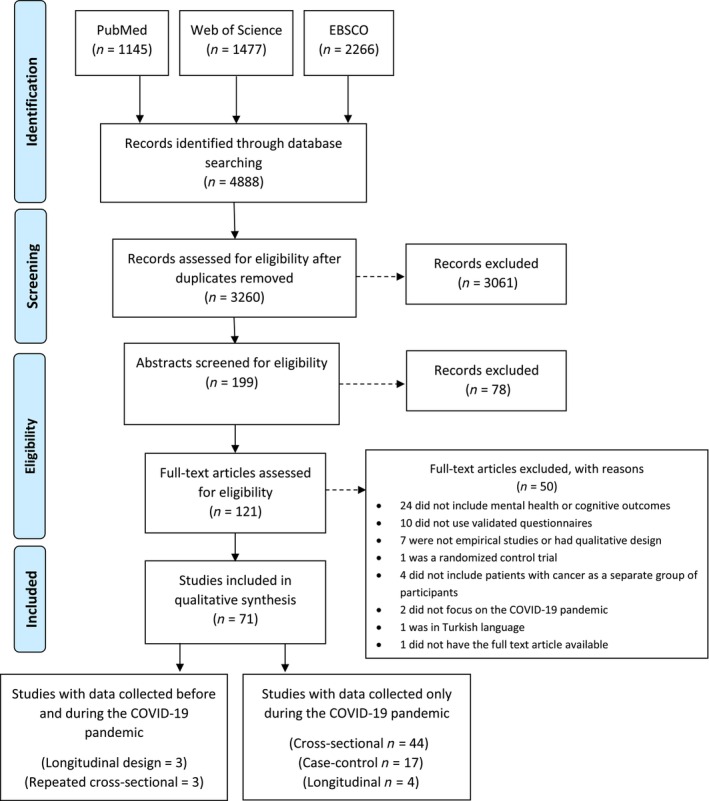
Flow diagram of study selection.

The search strategy yielded 3260 articles after removal of duplicates (see Figure 1). After an iterative review by title and abstract, 121 full text articles were retrieved and read in full. From those, 50 were eliminated for several reasons, resulting in 71 articles identified as relevant (Figure 1). Characteristics of the included studies, published between 2020 and 2022, are presented in Table 1 (cross‐sectional studies), in Table 2 (longitudinal and repeated cross‐sectional studies) and in Table 3 (case–control studies). From the 71 articles, 17 had a case–control design, 7 had prospective designs, and 47 had cross‐sectional designs. Geographically, 12 studies were performed in China; in the United States and in Turkey 7 studies; in Germany and in Italy 5 studies; in the United Kingdom 5 studies; in France 4 studies; in Denmark, Iran, Israel, and Spain 2 studies; in Australia, Bangladesh, Brazil, Canada, India, Iraq, Korea, Japan, Mexico, Egypt, Malaysia, Poland, Netherlands, Singapore, and South Africa a single study. There were three multicenter studies performed across several countries.

**TABLE 1:** Characteristics and main findings of the descriptive studies selected for the systematic review.

Study	SQ^a^	Design^b^	Study period^c^	*N*	Age (*M* ± SD)^d^	Gender (%W)^e^	Tumor site	Stage	Questionnaires	Cut‐off score	Outcomes	Main findings
Ahn et al. [34]	3	CS	15 July–15 August 2020 (M)	221	50.1 ± 13.3	76%	Mixed	Mixed	SAVE‐6 CAS	≥15 (mild anxiety) ≥5 (dysfunctional anxiety)	Anxiety to viral epidemics COVID‐19 anxiety and fear	48.8% of the sample reported general anxiety related to the COVID‐19 pandemic, and approximately 10% had dysfunctional anxiety.
Alrubai et al. [83]	3	CS	1 September–1 December 2020 (M)	200	ND	91.5%	Mixed	Mixed	DASS‐21	>9 (clinically significant depression) >7 (clinically significant anxiety)	Depression Anxiety	During the pandemic there were reports of high prevalence rates of symptoms of depression (22.0%) and anxiety (22.0%). A higher prevalence was found among those who had higher education and in younger patients.
Arrieta et al. [42]	3	LD	1 March 2020–28 February 2021 (E)	548	61.5 ± 12.9	56.9%	Thoracic	Mixed	DASS‐21	≥5 (clinically significant depression) ≥4 (clinically significant anxiety)	Depression Anxiety	A high proportion of patients with thoracic cancer reported high anxiety symptoms (30.5%) and depression (18%) during the COVID‐19 pandemic period.
Baffert et al. [44]	3	CS	May–June 2020 (E)	189	ND ± ND	60%	Mixed	Mixed	GAD‐7	≥15 (severe anxiety)	Anxiety	The proportion of patients with high anxiety levels during the COVID‐19 pandemic period was low (3.1%).
Bartmann et al. [61]	3	CS	April–June 2020 (E)	82	ND	100%	Breast	Mixed	DT	ND	Psychological distress	There were no significant differences in psychological distress associated with the COVID‐19 pandemic period, based on previous literature data of the same country.
Bäuerle et al. [23]	3	CS	16–30 March 2020 (E)	150	ND ± ND	52%	Mixed	Mixed	PHQ‐2 GAD‐2 DT	≥3 (major depressive symptoms) ND ≥5 (elevated psychological distress)	Depression Anxiety Psychological distress	16.7% reported significant levels of depressive symptoms, 20.7% clinically significant anxiety symptoms, and 54.7% had elevated levels of psychological distress. Elevated COVID‐19‐related fear was associated with higher depression, generalized anxiety symptoms and higher psychological distress.
Binswanger et al. [24]	3	CS	April–July 2020 (E‐M)	69	Median = 53	49%	Brain	Mixed	DT8	ND	Psychological distress	The proportion of patients who reported to be substantially distressed (30%) during the COVID‐19 pandemic is in line with reported data from non‐pandemic periods
Chapman et al. [79]	3	CS	9 April–26 May 2020 (E)	234	51 ± ND	100%	Breast	Mixed	FACT‐Cog HADS	ND ND	Cognition Anxiety Depression	Greater rumination as well as COVID‐19 related emotional vulnerability was associated with high levels of anxiety, depression and distress as well as poorer cognitive function.
Cheli et al. [81]	3	CS	June–November 2020 (E‐M)	1281	53.78 ± 13.36	66%	Mixed	Mixed	DASS‐21	ND	Psychological distress	Higher levels of distress were significantly related to higher levels of risk perception, lower levels of confidence in safeguards and high levels among all the personality traits.
Chen et al. [33]	3	CS	15–17 April 2020 (E)	326	ND	46.6%	Mixed	Mixed	SAS SDS	ND ND	Anxiety Depression	A total of 67.5% and 74.5% of the patients had anxiety and depression, respectively. Treatment delay or interruption, concerns about COVID‐19 and lung cancer, were the dominant factors contributing to such prevalence.
Chen et al. [45]	3	CS	14–21 March 2020 (E)	834	ND ± ND	100%	Breast	Mixed	PHQ‐9 GAD‐7	≥10 (moderate to severe depression) ≥10 (moderate to severe anxiety)	Depression Anxiety	The prevalence of depression and anxiety in cancer patients during the COVID‐19 pandemic was 20.6% and 15.5%, respectively. Medical comorbidities, living alone, deterioration of breast cancer, and affected treatment plan due to COVID‐19 pandemic were found to be risk factors for higher levels of depression and anxiety.
Choobin et al. [87]	3	CS	9–29 May 2020 (E)	139	45 ± 9.60	100%	Breast	Mixed	FACT‐Cog	ND	Cognition	COVID‐19 related emotional distress predicted subjective cognitive impairment in women with primary breast cancer.
Dahan et al. [82]	3	CS	12 May–12 June 2020 (E‐M)	242	57 ± ND	100%	Breast	Early	HADS	ND ND	Anxiety Depression	Psychological distress was significantly higher in patients under 50 years old, without a significant other, under remote work, with a history of depression and/or taking antidepressants or anxiolytics.
Dieperink et al. [26]	3	CS	May 2020 (E)	40	62.2 ± 13.2	65%	Mixed	Mixed	DT	ND	Psychological distress	Most patients did not experience severe psychological distress. 28% participants reported moderate to severe levels of distress.
Ellehuus et al. [46]	3	CS	22 May–13 June 2020 (E)	2236	67 ± 13.3	43%	Hematologic	Mixed	GAD‐7	≥5 (mild to severe)	Anxiety	During the pandemic period, the majority of the hematological cancer patients (79%) presented with low symptoms of anxiety, 16% had mild symptoms of anxiety, and 5% had moderate to severe symptoms of anxiety.
Esen [47]	3	CS	May–June 2020 (E)	218	52.89 ± 14.72	42.7%	Mixed	Mixed	GAD‐7	≥5 (mild to severe)	Anxiety	35.8% of the patients had clinically significant levels of anxiety.
Falcone et al. [88]	3	CS	18 March–4 April 2020 (E)	70	57 ± ND	57.1%	Mixed	Mixed	C‐19EIS	ND	COVID‐19 emotional impact	The emotional distress during the Covid‐19 outbreak was significantly higher in women and in patients under 65 years of age, but it is not associated with disease severity and stage.
Faro et al. [48]	3	CS	ND	61	62 ± 10.4	83.6%	ND	ND	PHQ‐2 GAD‐2	≥3 (clinically significant symptoms) ≥3 (clinically significant symptoms)	Depression Anxiety	24.6% and 26.2% of the patients met criteria for clinical anxiety and depression, respectively.
Frey et al. [35]	3	CS	30 March–13 April 2020 (E)	550	58 ± ND	100%	Ovarian	Mixed	CWS HADS‐A/‐D	ND ≥11 (clinically significant symptoms)	Cancer‐related worry Anxiety and depression	Young age, presumed immunocompromise and delay in cancer care were associated with higher levels of cancer worry, anxiety and depression.
Güç et al. [49]	3	CS	April–May 2020 (E)	761	58.0 ± 11.7	63.1%	Mixed	Mixed	STAI	≥44 (clinically significant symptoms)	Anxiety	44.2% of the patients presented clinically high levels of anxiety.
Gultekin et al. [36]	3	CS	1–30 May 2020 (E)	1251	55 ± ND	100%	Gynecological	Mixed	HADS	≥11 (clinically significant symptoms)	Anxiety Depression	Concern about not being able to visit the oncologist during the COVID‐19 pandemic and experiencing modifications of care due to the pandemic, were significantly associated with high anxiety levels. Having additional comorbidities and being concerned about not being able to visit the oncologist during the COVID‐19 pandemic, significantly contributed to high depression levels.
Helm, Kempski, and Galantino [20]	3	LD	18–24 May 2020 (E)	15	55.3 ± 9.8	100%	Breast	Mixed	DT	≥4 (clinically significant symptoms)	Psychological distress	The interruption of cancer rehabilitation services increased distress in breast cancer survivors.
Hill, Frost, and Martin [41]	3	CS	July–October 2020 (M)	100	55.03 ± 12.01	100%	Ovarian	Mixed	DASS‐21 FCOV	ND ND	Depression Anxiety Fear of COVID‐19	The fear of COVID‐19 was predictive of higher anxiety symptoms.
Juanjuan et al. [25, 30, 35, 37, 39, 40, 72, 75, 76, 83, 84, 85, 86, 95]	3	CS	16–19 February 2020 (E)	658	ND	100%	Breast	Mixed	GAD‐7 PHQ‐9 IES‐R	≥15 (severe anxiety) ≥15 (severe depression) ND	Anxiety Depression COVID‐19‐related distress	Severe levels of anxiety, depression and psychological distress were reported by 8.9%, 9.3%, and 20.8% of the patients, respectively. Poor physical condition and treatment discontinuation were found to be associated with higher psychological distress related to COVID‐19. Poor general condition, shorter duration after cancer diagnosis, aggressive breast cancer molecular subtypes, and close contact with patients with COVID‐19 were independent factors associated with anxiety. Poor general condition and central venous catheter flushing delay were factors that were independently associated with depression.
Karacin et al. [38]	3	CS	17 January–10 May 2020 (E)	110	Median = 63	63.6%	Mixed	II, III, IV	BAI	ND	Anxiety	Anxiety symptoms levels were higher in patients with COVID‐19 fear and anxiety.
Kamposioras et al. [54]	3	CS	18 May–1 July 2020 (E)	143	ND	17.9%	Colorectal	Mixed	GAD‐7	≥10 (moderate to severe anxiety)	Anxiety	5.5% of patients experienced moderate to severe anxiety. Anxiety levels were associated with concerns about contracting the infection and worries that COVID‐19 would affect their mental health and their experience of cancer care.
Lauricella et al. [56]	3	LD	11 March–4 May 2020 (E)	197	Median = 62	50%	Mixed	Mixed	DASS‐21 IES‐R	ND ND	Depression Anxiety COVID‐19‐related distress	There was a relatively small increase in depressive symptoms and anxiety from the start of the pandemic onwards.
Joly et al. [90]	3	CS	17 March–29 May 2020 (E)	734	64 ± 11.7	69%	Mixed	Mixed	FACT‐Cog IES‐R	ND ≥33 (clinically significant post‐traumatic symptoms)	Cognition COVID‐19‐related distress	21% of patients had post‐traumatic symptoms. Patients with this type of symptomatology had worse subjective cognitive functioning.
Massicotte, Ivers, and Savard [51]	3	CS	28 April–29 May 2020 (E)	36	53.6 ± 10.9	100%	Breast	Non‐metastatic	HADS	≥7 (clinically significant anxiety and depression symptoms)	Anxiety Depression	16.7% and 44.4% of the patients had significant depression and anxiety symptoms, respectively. About two‐thirds of the patients experienced specific stressors related to the pandemic and a higher level of concerns related to these stressors was significantly associated with higher levels of anxiety and depressive symptoms.
Miaskowski et al. [32]	3	CS	27 May–10 June 2020 (E)	187	63.3 ± 10.9	97.9%	Mixed	Mixed	IES‐R CES‐D STAI	≥24 (mild to severe stress symptoms) ND ND	COVID‐19‐related distress Depression Anxiety	31.6% of the patients had high levels of stress. These patients, in comparison with those who had lower scores, had a higher number of comorbidities, were fewer years from their cancer diagnosis, were more likely to report a diagnosis of depression, and had a lower functional status score.
Miaskowski et al. [86]	3	CS	27 May–10 September 2020 (E‐M)	606	62.7 ± 10.9	91.8%	Mixed	Mixed	CES‐D STAI	ND ND	Anxiety Depression	A high proportion of oncology patients experienced significant levels of loneliness during COVID‐19 that exceed previous benchmarks. All other symptoms severity scores were above the clinically meaningful cutoff scores, except evening fatigue. Predictors of loneliness were: being unmarried, having higher levels of social isolation, and higher depression and anxiety levels.
Mogami et al. [27]	3	CS	May 2020 (E)	34	ND ± ND	100%	Gynecological	Mixed	DT HADS	≥5 (clinically significant symptoms) ≥8 (clinically significant symptoms)	Psychological distress Anxiety Depression	General psychological distress and anxiety were higher in patients undergoing treatment than in patients doing follow‐up. While one third of the patients undergoing cancer treatment showed significantly high anxiety levels, 50% of patients showed high depression rates.
Patni et al. [52]	3	CS	1 June–6 July 2020 (E‐M)	294	51.3 ± 12.7	39.8%	Mixed	ND	GAD‐7 PHQ‐9	≥10 (moderate to severe anxiety) ≥10 (moderate to severe depression)	Anxiety Depression	Significant levels of anxiety and depression were noticed in 28.9% and 27.2% of the patients, respectively.
Rodrigues‐Oliveira et al. [28]	3	CS	June–August 2020 (E‐M)	50	58.8 ± 9.9	22%	Head and neck	Mixed	HADS DT	≥11 (clinically significant symptoms) ≥4 (clinically significant symptoms)	Anxiety Depression Psychological distress	22% patients had clinical levels of anxiety or/and depression. The pandemic did not seem to impact anxiety and depression levels negatively when compared with a historical control group. 42% of the patients had clinically significant psychological distress.
Romito et al. [21]	3	CS	2–29 April 2020 (E)	77	56.6 ± ND	49.4%	Lymphoma	Mixed	IES‐R HADS	≥24 (clinically significant post‐traumatic symptoms) ≥8 (clinically significant symptoms)	COVID‐19‐related distress Psychological distress Anxiety Depression	31% and 43% of the patients had significant depression and anxiety symptoms, respectively. A correlation between depression and distress related with COVID‐19 pandemic was observed.
Shinan‐Altman Levkovich, and Tavori [80]	3	CS	5–12 April 2020 (E)	151	51 ± ND	100%	Breast	Mixed	BSI	ND	Anxiety	Patients with higher anxiety levels had more contact with healthcare professionals but are more likely to cancel clinical appointments because of the COVID‐19.
Sigorski et al. [55]	3	CS	11–15 May 2020 (E)	612	Median = 63	54.6%	Mixed	Mixed	FCV‐19S STAI	ND ND	Fear of COVID‐19 Anxiety	The fear and anxiety in concern to SARS‐CoV‐2 are significantly lower than the anxiety associated with cancer and its consequences.
Swainston et al. [29]	3	CS	ND	234	51 ± 7.9	100%	Breast	Mixed	FACT‐Cog HADS	ND ND	Cognition Psychological distress Anxiety Depression	COVID‐19 related distress was associated with worse levels of anxiety, depression and cognitive functioning.
Toquero et al. [31]	3	CS	18 May–5 June 2020 (E)	104	62.0 ± ND	64.4%	Mixed	Mixed	HADS	≥10 (clinically significant psychological distress) ≥7 (clinically significant anxiety) ≥4 (clinically significant depression)	Psychological distress Anxiety Depression	52.8% of psychological distress, 42.3% of anxiety and 58.6% of depression rates were detected. The main factors related with higher rates of psychological symptomatology were history of treatment with psychotropics and the adoption of additional infection prevention measures because of the perception of high risk of severe COVID‐19 infection.
Turgeman et al. [50]	3	LD	March–May 2020 (E)	164	ND ± ND	56%	Mixed	Mixed	HADS	>8 (clinically significant symptoms)	Anxiety Depression	10% of the patients had significant levels of anxiety and 24% of depression initially but already in the pandemic period. Initially elevated COVID‐19 distress levels predicted patient anxiety, but not depression, after 1 month.
Wang et al. [39]	3	CS	9–19 April 2020 (E)	6213	50.6 ± 13.3	47.2%	Mixed	Mixed	GAD‐7 PHQ‐9	≥7 (clinically significant anxiety) >7 (clinically significant depression)	Anxiety Depression	The prevalence of depression and anxiety were 23.4% and 17.7%, respectively. Having a history of mental disorder, excessive drinking, and having higher levels of fatigue and pain were the factors associated with mental health problems in patients with cancer.
Wong et al. [53]	3	CS	23 April–26 June 2020 (E)	631	57.0 ± 11.2	72.4%	Mixed	Mixed	HADS	>8 (clinically significant symptoms) >8 (clinically significant symptoms)	Anxiety Depression	29% of the patients had clinically significant levels of anxiety, whereas a slightly lower proportion of 20.9% demonstrated depression symptoms.
Yang et al. [85]	3	CS	January–May 2020 (E)	197	36.5 ± 11.2	54.8%	Mixed	I, II	SAS	ND	Anxiety	Anxiety during the pandemic was positively associated with adverse childhood trauma and suicide ideation.
Yang et al. [84]	3	CS	August 2020–August 2021 (M‐L)	396	ND ± ND	34.3%	Mixed	Mixed	SAS SDS	>50 (clinically significant symptoms) >53 (clinically significant symptoms)	Anxiety Depression	In patients with cancer undergoing radiotherapy the incidence of anxiety and depression was 34.9% and 33.8%, respectively. Age, work status, educational level and clinical stage were related to anxiety and depression.
Yasin et al. [43]	3	CS	1 April–1 August 2020 (E‐M)	298	53.2 ± 10.8	100%	Breast	Mixed	STAI	>39 (high anxiety levels)	Anxiety	Almost half (48.3%) of the patients with breast cancer had high levels of anxiety. It was significantly higher in the metastatic cancer patients than the nonmetastatic group.
Yélamos Agua et al. [25]	3	CS	16–25 April 2020 (E)	2779	ND ± ND	86.9%	Mixed	Mixed	K‐6	>13 (severe)	Psychological distress	33.5% of the patients during the COVID‐19 pandemic reported clinically significant levels of psychological distress. Young patients with cancer presented significantly higher levels of distress.
Yousefi Afrashteh and Masoumi [89]	3	CS	26 September–15 November 2020 (M)	210	39.0 ± 12.4	100%	Breast	ND	BDI BAI	ND ND	Depression Anxiety	Patients who had less depression and anxiety scored higher in self‐compassion, that was partly associated with less death anxiety.
Zomerdijk et al. [30]	3	CS	22 July–19 August 2020 (M)	394	60.4 ± 12.8	47%	Hematologic	Mixed	K‐10	≥20 (mild to high)	Psychological distress	35% of hematological patients had high psychological distress levels. There are positive associations between perceived risk of contracting COVID‐19 and both psychological distress and unmet needs, with the association between perceived risk and distress stronger among patients that were single.

Abbreviations: CC = case–control; CS = cross‐sectional; LD = longitudinal; *N* = sample size; ND = not described in the study; RCS = repeated cross‐sectional. Questionnaires: 12‐GHQ = 12‐item General Health Questionnaire; BAI = Beck Anxiety Inventory; BDI = Beck Depression Inventory; C‐19EIS = Covid‐19 Emotional Impact Survey; CAS = Coronavirus Anxiety Scale; CES‐D = Center for Epidemiologic Studies Depression Scale; CWS = Cancer Worry Scale; DASS‐21 = Depression, Anxiety and Stress Scale—21 Items; DT = Distress Thermometer; FACT‐Cog = Functional Assessment of Cancer Therapy‐Cognitive Function; FCV‐19S = Fear of COVID‐19 Scale; GAD‐2 = Generalized Anxiety Disorder 2‐item; GAD‐7 = Generalized Anxiety Disorder 7‐item; HADS = Hospital Anxiety and Depression Scale; HADS‐A = subscale of Anxiety from de Hospital Anxiety and Depression Scale; HADS‐D = subscale of Depression from de Hospital Anxiety and Depression Scale; IES‐R = Impact of Event Scale‐(Revised); K‐10 = Kessler Psychological Distress Scale‐10 items; K‐6 = Kessler Psychological Distress Scale‐6 items; Mini‐MAC = Mini‐Mental Adjustment to Cancer Scale; PDI = Psychological Distress Inventory; PHQ‐2 = Patient Health Questionnaire 2‐item; PHQ‐8 = Patient Health Questionnaire 8‐item; PHQ‐9 = Patient Health Questionnaire 9‐item; PSWQ = The Penn State Worry Questionnaire; SAS = Zung Self‐rating Anxiety Scale; SAVE‐6 = Stress and Anxiety to Viral Epidemics‐6 items; SCL‐90 = Symptom Checklist 90; SDS = Self‐Rating Depression Scale; STAI = Spielberg State–Trait Anxiety Inventory; WHO‐5 = WHO‐Five Wellbeing Index.

^a^ The second column represents study's methodological quality (SQ), classified as strong (1), moderate (2), or weak (3). This assessment was based on the Quality Assessment Tool for Quantitative Studies (National Collaborating Centre for Methods and Tools, 2008).

^b^ The third column represents the study design.

^c^ The fourth column discriminates the recruitment period of each study. In parentheses, the studies are classified according to the phases of COVID‐19 during which they took place, namely (E) Early phase (January 2020–June 2020); (M) Middle phase (July 2020–December 2020); and (L) Late phase (January 2021–June 2022).

^d^ The sixth column presents the mean (*M*) age of the participants and the standard‐deviation (SD).

^e^ Gender is presented by percentage of women (%W).

**TABLE 2:** Main mental health outcomes differences between groups of patients with cancer before and during the COVID‐19 pandemic.

Study	SQ^a^	Design^b^	*N*	Age (*M* ± SD)^d^	Gender (%W)^e^	Tumor site	Stage	Questionnaires	Cut‐off score	Outcomes	Main findings
Amonoo et al. [59]	3	RCS Prepandemic (March 2019–January 2020) During pandemic (March 2020–January 2021)	124 81	54.2 ± 11.8 56.0 ± 11.6	62.9% 65.4%	Mixed	Mixed	HADS	ND	Anxiety Depression	No differences were found on anxiety or depression symptoms between the samples of patients with cancer before and during the COVID‐19 pandemic period.
Gallagher, Bennett, and Roper [57]	1	LD^c^ 1st assessment: 2017–2019 2nd assessment: April 2020	517	64.0 ± ND	54.4%	Mixed	Mixed	12‐GHQ	≥6 (clinically significant depressive symptoms)	Depression	Pre‐COVID‐19 depression and loneliness during the COVID‐19 crisis were significant predictors of depression during the pandemic.
Hulbert‐Williams et al. [60]	3	RCS Prepandemic (June–July 2019) During pandemic (June–July 2020)	41 103	57.6 ± 11.9 65.3 ± 9.4	62.9% 65.4%	Mixed	Mixed	DASS‐21	ND	Depression Anxiety	Anxiety was significantly lower after pandemic onset and depression levels remained stable.
Irusen et al. [58]	3	LD^c^ 1st assessment: 26 March 2020 2nd assessment: July–September 2020	60	63.2 ± ND	0%	Prostate	Localized	STAI	≥39 (clinically significant anxiety symptoms)	Anxiety	State anxiety decreased significantly from pre‐COVID‐19 to during COVID‐19 in men undergoing curative treatment for prostate cancer.
Nardone et al. [62]	3	RCS Prepandemic (May 2019) During pandemic (April 2020)	380 78	ND ± ND ND ± ND	52.4% 50%	Mixed	ND	BDI STAI DT	≥13 (clinically significant depressive symptoms) ≥40 (clinically significant anxiety symptoms) >4 (clinically significant psychological distress)	Depression Anxiety Psychological distress	Depression, anxiety and psychological distress in patients undergoing radiotherapy were significantly higher during the COVID‐19 outbreak when compared with data from the prepandemic period.
Yildirim, Poyraz, and Erdur [40]	3	LD^c^ 1st assessment: 3–22 February 2020 2nd assessment: 14 March–5 July 2020	595	50.5 ± 14.9	77.1%	Mixed	ND	BAI BDI	≥21 (clinically significant anxiety symptoms) ≥10 (clinically significant depressive symptoms)	Anxiety Depression	Depression and anxiety levels in patients with cancer were found to increase during the pandemic, and this increase was positively correlated with the disruption of their treatment.

Abbreviations: CC = case–control; CS = cross‐sectional; LD = longitudinal; *N* = sample size; ND = not described in the study; RCS = repeated cross‐sectional. Questionnaires: 12‐GHQ = 12‐item General Health Questionnaire; BAI = Beck Anxiety Inventory; BDI = Beck Depression Inventory; DASS‐21 = Depression, Anxiety and Stress Scale‐21 Items; DT = Distress Thermometer; HADS = Hospital Anxiety and Depression Scale; STAI = Spielberg State‐Trait Anxiety Inventory.

^a^ The second column represents study's methodological quality (SQ), classified as strong (1), moderate (2), or weak (3). This assessment was based on the Quality Assessment Tool for Quantitative Studies (National Collaborating Centre for Methods and Tools, 2008).

^b^ The third column represents the study design.

^c^ Longitudinal design study with data collected before and during the COVID‐19 pandemic.

^d^ The fifth column presents the mean (*M*) age of the participants and the standard‐deviation (SD).

^e^ Gender is presented by percentage of women (%W).

**TABLE 3:** Main mental health outcomes differences between patients with cancer and controls.

				Patients with cancer					Controls							
Study	SQ^a^	Design^b^	Study period^c^	*N*	Age (*M* ± SD)^d^	Gender (%fem)^e^	Tumor site	Stage	Control group	*N*	Age (*M* ± SD)	Gender (%fem)	Questionnaires	Cut‐off score	Outcomes	Main findings
Alagoz et al. [68]	3	CC	11–31 May 2020 (E)	234	55.6 ± 13.4	52.6%	Mixed	Mixed	General population excluding health care professionals	276	43.9 ± 11.9	44.9%	HADS‐A HADS‐D POMS	>10 (clinically significant symptoms) >7 (clinically significant symptoms) ND	Anxiety Depression Psychological distress	Patients with cancer were less likely to have psychological distress, anxiety, and depression symptoms compared with controls.
Bafunno et al. [69]	3	CC	April 2020 (E)	178	58 ± 14.4	51%	Mixed	Mixed	General population	86	42.3 ± 13.0	69.8%	HADS IES‐R	ND ND	Anxiety Depression COVID‐19‐related distress	Patients with cancer had lower levels of depression symptoms and psychological distress, but similar levels of anxiety, when compared with the general population.
Chaix et al. [75]	3	CC	31 March–7 April (E)	360	ND	ND	Breast	ND	Asthma Depression Migraine	497 459 455	ND	ND	PDI	≥14 (clinically significant symptoms)	Psychological distress	There was no significant difference between groups regarding psychological distress.
Guo et al. [22]	3	CC	26–19 February 2020 (E)	32	ND	ND	ND	ND	No chronic disease Other chronic disease	649 295	ND	ND	HADS‐A HADS‐D	≥8 (elevated anxiety) ≥8 (elevate depression)	Anxiety Depression	The rates of clinically significant anxiety and depression in patients with cancer, with other chronic disease and in participants without chronic diseases were 34.8%, 45.8%, and 31.5%, respectively.
Çölkesen et al. [74]	3	CC	10 April–15 May 2020 (E)	80	53.6 ± 12.2	45%	Mixed	Mixed	PID Severe Asthma CVS Disease HT DM Healthcare workers	80 80 80 80 80 80	38.9 ± 14.2 49.2 ± 13.8 59.6 ± 9.6 54.7 ± 9.5 52.2 ± 10.7 36.5 ± 7.3	55% 69% 51% 56% 49% 57%	HADS‐ A HADS‐D	>10 (clinically significant anxiety) >7 (clinically significant anxiety)	Anxiety Depression	Healthcare workers had higher levels of anxiety when compared with the other groups. There was no significant difference between the groups regarding depression. Among patients with cancer significant levels of anxiety and depression were found among 35% and 46.2%, respectively.
Han et al. [73]	3	LD	1st assessment: 14–24 February 2020 2nd assessment: 1–10 April 2020 3rd assessment: 15–25 May 2020	111	56.6 ± 9.6	49.5%	Mixed	Mixed	Family members	111	55.5 ± 10.3	56.8%	SCL‐90	ND	Psychological distress	Patients with cancer had higher levels of psychological distress than family members.
Lou et al. [63]	3	CC	3–11 April 2020 (E)	301	57.7 ± 12.3	84.1%	Mixed	Mixed	Healthy controls	242	52.8 ± 16.1	81.4%	GAD‐7 PHQ‐8	≥10 (clinically significant anxiety) ≥10 (clinically significant depression)	Anxiety Depression	Rates of generalized anxiety and depression were similar across groups. In particular, generalized anxiety and depression did not differ significantly between patients actively treated for cancer and healthy controls with no history of cancer.
Musche et al. [64]	2	CC	16–30 March 2020 (E)	150	ND	78%	Mixed	Mixed	Healthy controls	150	ND	74%	GAD‐7 DT	≥5 (mild to severe anxiety) ND	Anxiety Psychological distress	There were no significant differences regarding psychological distress and anxiety symptoms between patients with cancer and controls.
Ng et al. [67]	3	CC	28 April–3 May 2020 (E)	72	53 ± 8.3	ND	ND	ND	Healthy controls	45	57.8 ± 8.8	ND	HADS	ND	Psychological distress	Patients with cancer reported less psychological distress when compared with healthy controls.
Ng et al. [77]	3	CC	6–22 April 2020 (E)	624	57.2 ± 12.2	55.9%	Mixed	Mixed	Caregivers Health care workers	408 421	46.5 ± 13.3 35.9 ± 10.6	52.5% 73.9%	GAD‐7	≥10 (clinically significant anxiety)	Anxiety	The prevalence of clinically significant anxiety was 19.1%, 22.5%, and 14% for patients with cancer, their caregivers and health care workers, respectively.
Rentscher et al. [66]	3	CC	27 May–11 September 2020 (E‐M)	262	67.9 ± 5.3	100%	Breast	I, II, III	Matched general population	165	68.0 ± 6.0	100%	STAI CES‐D	ND ND	Anxiety Depression	There were no differences between patients and controls in depression and anxiety symptoms.
Steel et al. [78]	3	CC	April–May 2020 (E)	161	66.0 ± 11.1	61.9%	Mixed	0, I, II	Caregivers	78	64.5 ± 10.2	67.2%	GAD‐2 PHQ‐2	≥3 (Criteria for generalized anxiety disorder) ≥3 (Criteria for major depressive disorder)	Anxiety Depression	In the early months of the COVID‐19 pandemic, patients with cancer and their family caregivers subjectively reported increased levels of depression and anxiety. A total of 16.5% of the patients and 15.2% of the caregivers reported depressive symptoms, 18.4% and 19% reported anxiety.
Tasnim et al. [76]	3	CC	November 2020–January 2021 (M‐L)	10	ND ± ND	ND	ND	ND	Other medical conditions	961	ND ± ND	ND	GAD‐7 PHQ‐9	≥15 (moderate to severe anxiety) ≥15 (moderate to severe depression)	Anxiety Depression	Mean depression and anxiety scores were significantly higher among participants who reported having a cancer diagnosis.
Troschel et al. [71]	3	LD (12‐week timespan during the first COVID‐related lockdown)	22 April–15 July 2020 (E‐M)	63	48.3 ± 12.2	42.9%	Brain	Mixed	Family members	37	48.1 ± 14.9	78.4%	HADS‐A HADS‐D DT	ND ND ND	Anxiety Depression Psychological distress	Patients demonstrated increased depressive symptom burden when compared with immediate relatives. No significant differences were found in anxiety symptoms and psychological distress.
van de Poll‐Franse et al. [65]	3	CC	18 April–4 May 2020 (E)	4094	63 ± 11	39%	Mixed	Mixed	Gen. population	977	62.3 ± 13	39%	HADS‐A HADS‐D	ND ND	Anxiety Depression	There are no significant differences in anxiety and depression symptoms between patients with cancer and a matched group of the general population.
Yang et al. [70]	3	CC	17–19 April 2020 (E)	1106	ND	56%	Lymphoma	Mixed	Caregivers Gen. population	948 524	ND	69% 51%	SAS	>49 (mild to severe anxiety)	Anxiety	The prevalence of anxiety in patients with lymphoma and their caregivers was similar, but higher than in the general population.
Yang et al. [72]	3	CC	4–20 March 2020 (E)	609	ND	ND	ND	Mixed	Family members	421	ND	ND	SAS	>50 (clinically significant anxiety)	Anxiety	Patients and their relatives did not seem to differ in anxiety levels.

Abbreviations: CC = case–control; CS = cross‐sectional; CVS = cardiovascular system; DM = diabetes Mellitus; HT = hypertension; LD = longitudinal; *N* = sample size; ND = not described in the study; PID = primary Immunodeficiency disorder. Questionnaires: CES‐D = Center for Epidemiologic Studies Depression Scale; DT = Distress Thermometer; GAD‐2 = Generalized Anxiety Disorder 2‐item; GAD‐7 = Generalized Anxiety Disorder 7‐item; HADS = Hospital Anxiety and Depression Scale; HADS‐A = subscale of Anxiety from de Hospital Anxiety and Depression Scale; HADS‐D = subscale of Depression from de Hospital Anxiety and Depression Scale; IES‐R = Impact of Event Scale‐(Revised); PDI = Psychological Distress Inventory; PHQ‐2 = Patient Health Questionnaire 2‐item; PHQ‐8 = Patient Health Questionnaire 8‐item; POMS = Profile of Mood States; SAS = Zung Self‐rating Anxiety Scale; SCL‐90 = Symptom Checklist 90.

^a^ The second column represents study's methodological quality (SQ), classified as strong (1), moderate (2), or weak (3). This assessment was based on the Quality Assessment Tool for Quantitative Studies (National Collaborating Centre for Methods and Tools, 2008).

^b^ The third column represents the study design.

^c^ The fourth column discriminates the recruitment period of each study. In parentheses, the studies are classified according to the phases of COVID‐19 during which they took place, namely (E) Early phase (January 2020–June 2020); (M) Middle phase (July 2020–December 2020); and (L) Late phase (January 2021–June 2022).

^d^ The sixth column presents the mean (*M*) age of the participants and the standard‐deviation (SD).

^e^ Gender is presented by percentage of women (%W).

All studies combined included 35 752 patients with cancer (range 10–6213) aged over 18 years, and most participants were women. Regarding characteristics of cancer, while most studies did not differentiate between cancer types based on location, 14 were conducted in patients with breast cancer, 4 in patients with gynecological and ovarian cancers, 4 in lymphoproliferative and hematologic cancers, 2 in patients with brain tumors, 1 in head and neck cancers, 1 in thoracic cancers, 1 in colorectal cancers, and 1 in prostate cancer. Regarding the risk of bias, the majority of the studies had a high risk of selection bias or did not describe the selection of study participants adequately. Only 17 studies included at least one control group to compare mental health variables between patients with cancer and the general or specific groups of the population, none of which had cognition as an outcome (Table 3). Furthermore, we found only three longitudinal studies with data collected prior to and during the COVID‐19 pandemic, as well as three repeated cross‐sectional studies with assessments conducted before and during the pandemic (Table 2). This limited number of longitudinal and repeated cross‐sectional studies, combined with their diverse methodologies and outcome measures, posed significant challenges for aggregating data with meta‐analyses.

### 3.2 Measurements of Mental Health, Depression, and Anxiety Symptoms

Only studies using validated measures were included. In all articles, mental health outcomes were self‐reported. The Hospital Anxiety and Depression Scale (HADS) was the most used scale to assess general psychological distress, with its subscales used to assess depression and anxiety symptoms (20 articles). To measure symptoms of depression, the following were also used: Self‐Rating Depression Scale (SDS); 12‐item General Health Questionnaire (12‐GHQ); Patient Health Questionnaire 2/8/9‐item (PHQ‐2/8/9); Beck Depression Inventory (BDI‐II); Depression, Anxiety and Stress Scale (DASS‐21); Profile of Mood States (POMS); Center for Epidemiologic Studies Depression Scale (CES‐D). The Beck Anxiety Inventory (BAI), Brief Symptom Inventory—Anxiety subscale (BSI), Generalized Anxiety Disorder 2/7‐item (GAD‐2/7), Spielberg State‐Trait Anxiety Inventory (STAI), Zung Self‐rating Anxiety Scale (SAS), DASS‐21 and POMS were also used to evaluate symptoms of anxiety. Finally, other scales used to measure psychological distress were Psychological Distress Inventory (PDI); Symptom Checklist 90 (SCL‐90); Kessler Psychological Distress Scale (K‐6/10); Distress Thermometer (DT). Symptoms of distress caused specifically by the COVID‐19 outbreak were assessed by the following: Impact of Event Scale‐Revised (IES‐R); COVID‐19 anxiety scale (CAS); Fear of COVID‐19 Scale (FCOV); Covid‐19 Emotional Impact Survey (C‐19EIS); Stress and Anxiety to Viral Epidemics‐6 items (SAVE‐6). Cognitive function was assessed in four studies with the Functional Assessment of Cancer Therapy‐Cognitive Function (FACT‐Cog).

### 3.3 Study Quality

Methodological quality assessment of the included studies is presented in Tables 1, 2, 3, based on participant's selection bias, study design, presence of confounders, blinding, and data collection methods. The global quality of the studies included was considered weak in most cases, with only one study having a moderate methodological quality and one study graded as high quality.

### 3.4 Prevalence of Psychological Distress, Anxiety, and Depression Symptoms Among Patients With Cancer During Temporal Exposure to the COVID‐19 Pandemic

Prevalence of depression, anxiety, and general psychological distress was quite variable among the included studies. In addition to use of several measures, different cut‐off points were often considered to identify patients with psychological distress, anxiety, and depression. Most studies showed high levels of psychological distress, with prevalence rates between 28% and 58% among patients [20, 21, 22, 23, 24, 25, 26, 27, 28, 29, 30, 31, 32]. Significant symptoms of depression ranged from 4.2% to 74.5% of patients, while significant anxiety levels were found in 3.1%–51.4% of the participants in the included studies [20, 21, 29, 33, 34, 35, 36, 37, 38, 39, 40, 41, 42, 43, 44, 45, 46, 47, 48, 49, 50, 51, 52, 53, 54]. Importantly, a study comparing anxiety specifically related to the COVID‐19 pandemic to anxiety associated with cancer found that COVID‐related fear and anxiety were significantly lower [55]. Regarding changes across the pandemic, a longitudinal study with data collected at the start of the pandemic and approximately 7 months later showed only a small increase in depression and anxiety symptoms [56].

### 3.5 Pre‐ to Post‐COVID‐19 Pandemic Changes in Psychological Depression Symptoms

Among the included studies in the review, three of them had a longitudinal design with data collected in the period prior to onset of the COVID‐19 pandemic, and data obtained during the pandemic (Table 2). Gallagher, Bennett, and Roper [57] found that having depression prior to COVID‐19 was a significant predictor of depression during the pandemic. Another longitudinal study, by Irusen et al., using a sample comprised by patients with prostate cancer undergoing curative treatments, found that the COVID‐19 period did not induce significant levels of anxiety. In fact, these authors described a significant reduction in state anxiety during the pandemic [58]. Finally, Yildirim, Poyraz, and Erdur [40], in a longitudinal study using a sample of patients with breast, ovarian, colon, and gastric cancer, showed that while depression and anxiety levels were found to increase during the pandemic, this was specifically associated with disruptions of the cancer treatment plan.

Albeit not having a longitudinal design, three repeated cross‐sectional studies considered differences in affective symptoms between groups of patients from the prepandemic period and patients exposed to the COVID‐19 pandemic (Table 2). Two studies including patients with different cancer types and disease stages did not find significant differences in severity of depression symptoms depending on the period of assessment (pre vs. during COVID‐19) [59, 60]. Regarding anxiety, these two studies had distinct findings: while no differences were found in the study of Amonoo et al. [59], Hulbert‐Williams et al. [60] showed that during the COVID‐19 period patients with cancer had lower anxiety levels when compared with patients assessed in the prepandemic period. Although these two studies have quite different proportions of patients included in the prepandemic period (larger in the study of Amonoo et al.) and during the COVID‐19 (larger in the study of Hulbert‐Williams et al.), their findings are globally consistent with those reported by Bartmann et al., who described levels of psychological distress in a small sample of patients with breast cancer that were similar to prepandemic data available in the same country [61]. Conversely, one study that included patients undergoing radiotherapy treatment (regardless of cancer type) found higher prevalence rates of anxiety, depression, and general psychological distress among patients assessed during the COVID‐19 outbreak period [62].

### 3.6 Mental Health Outcomes During COVID‐19 Pandemic in Patients With Cancer in Comparison With General Population and Subgroups

The systematic review included several studies using different populations as control groups, as described in Table 3, namely the general population, caregivers, family members, healthcare workers, and other clinical samples. Using samples of patients with various types of tumors and stages in comparison with the general population and healthy controls, four studies reported no significant differences in levels of psychological distress, anxiety, and depression between these groups [63, 64, 65, 66]. However, these findings were not consistent with those of other studies. One study, with a smaller sample size, showed that patients with cancer reported less psychological distress, anxiety, and depression symptoms during the pandemic compared with healthy controls [67], which was corroborated by Alagoz et al., with larger sample sizes [68], and by Bafunno et al. [69]. The latter showed lower levels of depression and psychological distress, and similar levels of anxiety, in patients with cancer compared with the general population [69]. This tendency was not reproduced in another study with a more specific sample of patients with lymphoma, reporting higher levels of anxiety symptoms in patients with cancer when compared with the general population, but similar levels of symptoms when compared with their caregivers [70].

In other comparisons with relatives, one study found that patients with cancer had higher levels of depression symptoms [71]. This effect was not found for anxiety, where differences between patients with cancer and their relatives were not found [71, 72]. Regarding psychological distress, evidence from the selected studies is not consistent. Using different measures to assess the construct, Han et al. [73] found that patients had higher levels of psychological distress, while Troschel et al. did not find significant differences between the two groups [71] Finally, in comparisons with healthcare workers, patients with cancer were described to have similar levels of depression symptoms, but lower levels of anxiety [74].

Furthermore, when compared with patients with other chronic diseases, namely asthma, migraine, depression, primary immunodeficiency disorder, cardiovascular disease, hypertension and diabetes, patients with cancer showed similar levels of psychological distress [74, 75]. On the contrary, Tasnim et al. [76], using a small sample comprised by 10 patients with cancer, showed higher levels of anxiety and depression symptoms when compared with patients with other medical conditions (*n* = 961). However, caution is advised in interpreting these results due to the small sample and risk for sampling bias. Finally, we also note that, in three of the controlled studies [22, 77, 78], only the percentage of participants with clinically significant anxiety and psychological distress was provided for each group (Table 3), precluding statements regarding more detailed comparisons between groups.

### 3.7 Factors Associated With Mental Health Outcomes During the COVID‐19 Pandemic

The evidence consistently showed that COVID‐19 fear and anxiety, along with greater rumination and COVID‐related emotional vulnerability, were associated with higher levels of general anxiety, depression, and psychological distress [23, 29, 34, 38, 41, 79]. Positive correlations were also found between levels of anxiety and depression in patients with cancer and worry about not being able to visit the oncologist, or of experiencing treatment disruptions, or of delays in cancer care, due to the COVID‐19 pandemic [33, 35, 36, 37, 40, 45, 51, 54, 72]. Anxiety levels, in particular, were notably linked to several COVID‐19‐related concerns. Many patients were anxious about contracting the virus and worried that COVID‐19 would negatively impact their mental health and overall cancer care experience [54]. Interestingly, those with higher anxiety levels tended to have more frequent contact with healthcare professionals, yet they were also more likely to cancel clinical appointments due to fears related to the pandemic [80]. In addition, treatment disruptions and deep concern about COVID‐19 seemed to be associated with a heightened fear of disease progression [33]. Close contact with patients with COVID‐19 and perceived risk of contracting COVID‐19 were associated with higher levels of anxiety and psychological distress [81], but not with depression [30, 37, 50]. Importantly, a study conducted in Poland found that for patients with cancer under actively treatment, cancer‐related consequences were more feared than the risk of contracting SARS‐CoV‐2 infection [55].

Demographic and personal factors also played significant roles in the levels of distress. Some studies found that younger and socially isolated patients had higher levels of distress [20, 82]. Specifically, regarding anxiety and depression symptoms, a positive association was found with age below 65 years, being single, having a higher education, a history of mental disorder, excessive drinking habits, not having a professional occupation, high levels of fatigue and pain, disruption of cancer treatment, and being self‐described as immunocompromised [25, 30, 35, 39, 40, 83, 84]. Anxiety symptoms, in particular, were associated with female gender, younger age, the presence of suicidal ideation, and a history of adverse childhood trauma [21, 85]. Gallagher, Bennett, and Roper [57] found that loneliness experienced during the COVID‐19 pandemic was an important predictor of depression in patients with cancer, with a positive correlation between loneliness and higher levels of depression and anxiety also found in a cross‐sectional study by Miaskowski et al. [86].

Specifically in breast cancer, high rates of depression, anxiety, and insomnia were observed in patients with poor general condition, with a shorter interval after cancer diagnosis and aggressive breast cancer molecular subtypes associated with higher levels of anxiety [37]. Patients with breast cancer and with medical comorbidities, living alone, with low social support, difficulty obtaining essential goods during the pandemic period, or experiencing disease progression, had higher levels of anxiety and depression [45, 51, 87]. While a study conducted by Falcone et al. suggested that disease severity and stage were not significantly associated with anxiety and depression symptoms [88], a more recent study showed that cancer clinical stage was related to this type of symptoms [84]. Finally, also in women with breast cancer, a perceived threat to job security was found to be positively associated with depression [79]. Importantly, regarding possible protective factors, a study by Yousefi Afrashteh and Masoumi suggested that during the pandemic, self‐compassion in patients with breast cancer may buffer the symptoms of anxiety and depression [89].

### 3.8 The Impact of COVID‐19 on the Cognitive Function of Patients With Cancer

Cognitive function was assessed in four studies with subjective self‐report measures, namely using the FACT‐Cog. Three studies in women with breast cancer found a negative correlation between subjective cognitive function and COVID‐related emotional distress and vulnerability as well as with rumination [29, 79, 87]. Consistently, the only study including a sample of patients with mixed types of cancer and stages showed that those with posttraumatic symptoms had worse subjective cognitive functioning [90].

## 4 Discussion

This systematic review summarizes 71 studies, published up to February 2022, addressing the relationship between the COVID‐19 pandemic and mental health and cognition outcomes in patients with cancer. In general, our review suggests that although many studies reported high levels of psychological distress, anxiety, and depression, longitudinal studies did not find consistent differences between the pre‐pandemic and the COVID‐19 pandemic periods. Importantly, there were also no consistent differences in mental health status between patients with cancer and other populations during the COVID‐19 pandemic period. Cognitive complaints during the pandemic were reported to be associated with COVID‐19‐related emotional distress and vulnerability.

Prevalence rates of depression, anxiety, and psychological distress were very different between studies. Indeed, studies were conducted in different countries and were mostly cross‐sectional, with data collected at very different times, namely regarding the moment of the pandemic in that specific site. It is possible that levels of psychological distress, anxiety, and depression could have varied at different times of the pandemic, depending for instance of COVID‐19‐related parameters such as number of new cases, new deaths, and government‐imposed stringency measures. Indeed there is some evidence that, in the general population, the prevalence of clinically significant depressive symptoms was significantly lower in countries where governments implemented stringent policies promptly [91]. Additionally, it is important to consider the significant heterogeneity of questionnaires employed in the studies to assess the outcomes of interest, sometimes with different cutoff points being used to identify patients with significant emotional distress, anxiety, or depression.

Importantly, even if they were rare, the longitudinal studies included in this systematic review did not support a clear mental health impact of the COVID‐19 pandemic in patients with cancer. While Yildirim, Poyraz, and Erdur [40] showed an increase in depression and anxiety levels during the pandemic, these symptoms seemed to be associated with disruption of the cancer treatment plan, suggesting that the main concern of patients, even in times of crisis, continues to be cancer and everything that may have an impact on its course. Paradoxically, another longitudinal study with data collected in a slightly later phase of the pandemic (July–September 2020) [58] showed a decrease in anxiety symptoms. This finding may suggest an acute increase in affective symptoms at the onset of the pandemic followed by a trend to decrease over time as patients adapted. Supporting this hypothesis, there is some evidence in the general population suggesting emotional adaptation with a decrease in anxiety and depression after the initial weeks of COVID‐19 [92]. The results from the three repeated cross‐sectional studies included in this review are in line with the available longitudinal data. Only the study that collected data at an earlier stage of the pandemic (April 2020) suggested that depression, anxiety, and psychological distress were higher in patients assessed during the pandemic in comparison with patients assessed prior to the pandemic [62]. Importantly, this study was conducted with patients undergoing radiotherapy, which is a particularly vulnerable population, often with underdiagnosed and untreated anxiety and depression [93, 94]. Moreover, the results from the two other repeated cross‐sectional studies did not find differences in depressive symptoms between patients assessed prior to the pandemic and those assessed during the pandemic period [59, 60]. In fact, one of them [60] showed that patients assessed during the pandemic had significantly lower levels of anxiety, which aligns with longitudinal findings in Irusen et al. [58].

While it seems unquestionable that patients with cancer faced challenges regarding their mental health during the COVID‐19 pandemic, it remains unclear if they were at higher risk in comparison with other groups of the population. When compared with the general population, all of the included studies were consistent in showing similar [63, 64, 65, 66], or even lower [67, 68, 69], symptoms of depression and psychological distress in patients with cancer. Regarding anxiety, while one study described higher levels in comparison with healthy controls [70], studies including patients with mixed types and stages of cancer did not find significant differences [63, 64, 65, 66, 69]. Paradoxically, in one study, excluding health care professionals from the general population sample, patients with cancer reported lower anxiety levels [68]. However, in the only study reporting a comparison with healthcare workers, patients with cancer reported similar levels of affective symptoms [74], while comparisons with relatives suggested higher levels of depression, but not anxiety [71, 72]. Finally, the majority of the studies with control groups comprised by other chronic diseases found similar levels of anxiety and depression in patients with cancer [75, 76]. Despite the inconsistent results between studies, in a recent systematic review, Xiong et al. [95] showed that the COVID‐19 pandemic was associated with significant levels of psychological distress in the general population, with prevalence rates in line with our review. Our findings do not challenge the suggestion that the pandemic represented an unprecedented thread to mental health, but do not support that patients with cancer were particularly affected relative to the general population.

This review has also identified some variables associated with poor mental health outcomes during the COVID‐19 pandemic in patients with cancer. Among the pandemic‐related factors, fear, anxiety, rumination, and emotional vulnerability linked to the COVID‐19 outbreak were associated with greater psychological distress, anxiety, and depression across studies, irrespective of the cancer site and stage [23, 29, 32, 33, 34, 38, 41, 79]. Some of these variables were also found to be associated with psychological status during the pandemic in the general population [96, 97]. On the other hand, factors related with concern about the impact of the pandemic on cancer care were also associated with increased levels of anxiety and depression, namely worry about treatment disruptions and delay, which were also associated with fear of disease progression [33, 35, 36, 37, 40, 45, 51, 54, 72]. Anxiety symptoms, in particular, were found to be linked to concerns about contracting COVID‐19, worries about its impact on mental health and cancer care, increased contact with healthcare professionals, and a higher likelihood of cancelling appointments [54, 80]. These findings are consistent with previous literature, which has shown that increased anxiety can lead to avoidance behaviors, such as cancelling or delaying medical appointments, a common response among individuals experiencing health anxiety [3, 98]. The increased contact with healthcare professionals despite high anxiety levels reflects a heightened need for reassurance and support, a pattern observed in other studies exploring the psychological impact of COVID‐19 on patients with chronic illnesses [39]. Interestingly, these findings collectively suggest that the cancer diagnosis remained the main source of concern of these patients. This is consistent with the study by Sigorski et al. [55], which showed that fear and anxiety related to SARS‐CoV‐2 were significantly lower than the anxiety associated with cancer and its consequences. Consistently, cancer‐related factors, such as having an aggressive molecular cancer subtype and a shorter duration after cancer diagnosis, are particularly associated with anxiety symptoms [33, 36]. In addition, some individual and social‐level characteristics were also shown to be associated with worse mental health outcomes. Young age [35, 82], being socially isolated and having low perceived available resources [82], having a history of a mental disorder, excessive drinking habits, feelings of fatigue and pain [39], self‐describing as immunocompromised [35], and having a poor general condition [37] were associated with higher psychological distress. In a longitudinal study, previous depression and experiencing loneliness were predictors of depression during the COVID‐19 pandemic in patients with cancer [57], which is consistent with findings in the general population, both during the COVID‐19 pandemic [99, 100] and in research conducted prior to the pandemic [101, 102]. Additionally, anxiety during the pandemic was associated with adverse childhood trauma and suicidal ideation [85]. This finding is consistent with previous research highlighting the long‐term impact of early life stressors on mental health. Studies with the general population have shown that individuals with a history of childhood trauma are at a higher risk for developing anxiety disorders and suicidal ideation, particularly during times of increased stress such as the COVID‐19 pandemic [103, 104].

Unfortunately, this review did not allow for precise conclusions about the impact of the COVID‐19 pandemic period on the cognitive function of oncological patients. We did not find any study assessing cognitive function with objective neuropsychological validated tests. However, we found four studies, three of them in patients with breast cancer, assessing subjective cognitive function with the FACT‐Cog, a self‐reported measure of cognitive complaints [29, 79, 87, 90]. Patients reported poorer subjective cognitive functioning when they perceived the pandemic as a threat to job security and when having greater COVID‐related distress and rumination. However, these results seem to be influenced by the presence of anxiety of depression that often implies subjective cognitive deficits [105, 106].

### 4.1 Study Limitations

Our review is strengthened by several methodological procedures to reduce several sources of bias. We minimized database and scope bias by using three reference databases, and including publications in multiple languages, respectively. Inaddition, researchers with multiple backgrounds (psychology, neuropsychology, psychiatry, and/or oncology) were involved. Nevertheless, this systematic review is not free of limitations. First, the description of findings from each study was narrative and qualitative, which does not directly address our main aim of assessing changes in mental health outcomes from pre to during the pandemic period. Second, the vast majority of these studies were cross‐sectional, limiting inference about associations. Only three of the included studies had a longitudinal design, with data collected before and during the COVID‐19 crisis, one of which did not report scores to allow for longitudinal comparisons of continuous data. As a result, we were unable to perform a meta‐analysis to answer our primary research question comprehensively. Due to the limited number of longitudinal studies, we adapted our methodology to include cross‐sectional and case–control studies to ensure a comprehensive analysis. This adaptation, while necessary to capture a broader range of data, may limit the ability to draw firm conclusions about changes over time. However, by including these additional studies, we were able to provide a more detailed and comprehensive overview of the impact of the COVID‐19 pandemic on mental health and cognitive outcomes in patients with cancer. Third, many studies collected their data online which, while unavoidable given the measures imposed to contain the spread of the SARS‐COV‐2 virus, may have jeopardized the quality and generalizability of findings. Finally, considering the heterogeneity of cancer stages and types in the studies, as well as of the time from diagnosis to study enrolment and the precise timing of assessment during the pandemic, could have affected the results. Therefore, the current review is far from precisely enabling the assessment of the full impact of the COVID‐19 pandemic, and more detailed longitudinal studies are needed to reach more definitive conclusions. Despite these limitations, the systematic and rigorous approach we employed enhances the robustness of our findings and provides valuable insights into the mental health and cognitive challenges faced by patients with cancer during the pandemic.

### 4.2 Clinical Implications

An important finding of our systematic review was the impact of treatment interruptions as a significant stressor for patients with cancer during the COVID‐19 pandemic. Treatment delays and interruptions were prevalent due to the reallocation of healthcare resources, lockdown measures, and the need to minimize exposure risk. These disruptions not only exacerbated psychological distress in patients but had potential impact on cancer‐related outcomes. For instance, the study conducted by Kamposioras et al. [54] highlighted that the fear of contracting COVID‐19 led to delays in seeking care, resulting in treatment interruptions, which is consistent with other studies included in this review [40, 41]. Moving forward, it is imperative for health systems to develop robust contingency plans to ensure the continuity of cancer care during future health crises. This includes establishing protocols for safe treatment delivery, leveraging telemedicine for consultations, and ensuring clear communication with patients about the importance of maintaining treatment schedules. Such processes can mitigate the psychological and clinical impact of treatment disruptions on patients with cancer, in the face of unforeseen global health emergencies. While further quantitative research is needed to clarify these findings, we believe that available data support preventive and supportive measures for mental health care in patients with cancer to be conducted during the pandemic period as in its absence, since cancer‐related consequences remain the main determinant factor for mental wellbeing.

### 4.3 Conclusions

This systematic review provides evidence about mental health outcomes, namely psychological distress, depression, and anxiety, as well as subjective cognitive function, in patients with cancer during the COVID‐19 pandemic. It also provides an outlook on how these outcomes are related to individual and pandemic‐related factors, and how they compare with other population groups. High prevalence of psychological distress, anxiety, and depression symptoms was reported in most studies, and may have been associated with fears of disruption of oncological care. However, while Covid‐19‐related fear and perceived risk were reported as important contributing factors to anxiety and psychological distress, the few existing longitudinal studies did not consistently report a significant increase of affective symptoms relative to before the pandemic. Additionally, there was no consistent evidence of increased psychological vulnerability relative to other groups, namely the general population, with most studies showing that levels of anxiety and depression symptoms were similar or lower in patients with cancer during this period. Overall, while this is supportive of limited impact of the COVID‐19 pandemic on mental health outcomes in patients with cancer, additional high‐quality longitudinal studies are necessary to address this question.

## Author Contributions

Conceptualization and methodology: S.A., D.F., and A.J.O.‐M. Formal analysis and investigation: D.F. and S.A. Data curation: D.F., S.A., and A.J.O.‐M. Writing – original draft preparation: D.F., S.A., and A.J.O.‐M. Writing – review and editing: D.F., S.A., M.T.C., T.N., B.S., F.C., and A.J.O.‐M. Supervision: F.C. and A.J.O.‐M. All authors have read and agreed to the published version of the manuscript.

## Supporting information


Data S1.

## Data Availability

All data analyzed during this study are included in this published article [and its supplementary information files]. No additional datasets were used or created during the current study.
